# Pathogenicity and virulence of *Mycobacterium tuberculosis*

**DOI:** 10.1080/21505594.2022.2150449

**Published:** 2023-01-04

**Authors:** Kathryn C. Rahlwes, Beatriz R.S. Dias, Priscila C. Campos, Samuel Alvarez-Arguedas, Michael U. Shiloh

**Affiliations:** a Department of Internal Medicine, University of Texas Southwestern Medical Center, Dallas, TX, USA; b Department of Microbiology, University of Texas Southwestern Medical Center, Dallas, TX, USA

**Keywords:** *Mycobacterium tuberculosis*, tuberculosis, virulence factors, infectious disease, host–pathogen interactions

## Abstract

*Mycobacterium tuberculosis* (Mtb) is the causative agent of tuberculosis, an infectious disease with one of the highest morbidity and mortality rates worldwide. Leveraging its highly evolved repertoire of non-protein and protein virulence factors, Mtb invades through the airway, subverts host immunity, establishes its survival niche, and ultimately escapes in the setting of active disease to initiate another round of infection in a naive host. In this review, we will provide a concise synopsis of the infectious life cycle of Mtb and its clinical and epidemiologic significance. We will also take stock of its virulence factors and pathogenic mechanisms that modulate host immunity and facilitate its spread. Developing a greater understanding of the interface between Mtb virulence factors and host defences will enable progress toward improved vaccines and therapeutics to prevent and treat tuberculosis.

## Introduction

Tuberculosis (TB) is a contagious infectious disease caused by the bacillus *Mycobacterium tuberculosis* (Mtb), a remarkably successful pathogen that primarily infects the lungs, leading to the classic syndrome of pulmonary TB. In addition, all other organs and tissues including the lymph nodes, brain, kidneys, and spine can be affected in a disorder called extrapulmonary TB [1]. According to the World Health Organization (WHO) Global TB Report 2021 [2], approximately 25% of the world’s population has immunologic evidence of prior infection with Mtb as determined by surveillance testing, and in 2020, 10 million people developed the active form of tuberculosis (ATB). TB represents one of the deadliest infections in the world, and, along with malaria and HIV/AIDS, has had the most significant socioeconomic impact on humanity [3]. The most up-to-date mortality data indicate that in 2020, 1.4 million people died of TB, thus representing the second leading infectious cause of death globally after COVID-19 [2,4]. While many of these cases and deaths arise from primary tuberculosis occurring after an initial infection, latent TB infection (LTBI), in which the bacteria can remain quiescent for decades from the initial exposure without causing TB disease [4–6], also accounts for a significant proportion of ATB. Indeed, 5–10% of individuals with LTBI subsequently develop ATB, and the risk of converting from latent to active disease increases substantially when individuals experience other medical conditions such as cancer, HIV/AIDS, diabetes, kidney failure, and viral coinfections such as with coronavirus. Children under 5 years of age are also at high risk of progression from LTBI to ATB [7,8].

For a microbe isolated and identified nearly 150 years ago that has caused immeasurable suffering and death, it is remarkable that an effective vaccine is not available. While vaccination with the Bacillus Calmette–Guérin (BCG) – the attenuated vaccine derived from *M. bovis* – protects against disseminated forms of TB in children, it does not prevent primary infection and does not provide consistent protection against infection in adults. Thus, the search for an improved vaccine able to prevent childhood and adult pulmonary TB remains critical [9,10]. Recent work demonstrating that intravenous vaccination of macaques with BCG afforded the animals a superior immune response and marked protection against subsequent airway infection with Mtb provides compelling evidence that the mammalian immune system can be harnessed to prevent Mtb infection [11,12].

As previously stated, a vast number of people demonstrate evidence of past or ongoing infection with Mtb. Currently, there are several methods of diagnosis, depending on whether active or latent TB is suspected. For diagnosis of LTBI or concern for ATB, current immunologic methods include the Mantoux Tuberculin skin test (TST) and whole-blood interferon-gamma (IFN-γ) release assays (IGRAs) [13]. Both assays test for the presence of an amnestic cell mediated immune response. TST is an *in vivo* test performed through a subcutaneous injection of an antigen (tuberculin or purified protein derivative) in the lower part of the arm of the subject, who must return within 48–72 hours for determination of induration of the skin at the site of injection. While TST is an affordable, simple method to screen and detect the presence of cell mediated immunity to Mtb, it can also yield false-positive results in people vaccinated with BCG or false-negative results in immunosuppressed individuals [8,14,15]. IGRAs are blood tests that measure levels of IFN-γ following *in vitro* stimulation of human lymphocytes with two Mtb virulence factors – the early secreting antigen target-6 (ESAT-6, EsxA) and culture filtrate protein-10 (CFP-10, EsxB). IGRAs demonstrate improved accuracy, sensitivity, and specificity in detecting Mtb infection, and in addition are not affected by prior BCG vaccination since the BCG strain lacks the EsxA and EsxB antigens and thus individuals vaccinated with BCG (but not infected with Mtb) should have no immunologic memory of exposure to EsxA and EsxB [16,17]. However, neither TST nor IGRAs can distinguish between ATB and LTBI or predict the risk of developing ATB in people with LTBI [18,19]. At the present time, treatment of individuals with LTBI (i.e. with no symptoms or clear signs of developing ATB) is recommended in order to reduce the risk of progression to ATB, especially in populations with a high risk of reactivation such as recent LTBI converters, immunocompromised patients living with HIV/AIDS, health care workers, and patients starting tumor necrosis factor-alpha (TNF-α) inhibitors [20,21]. Research in this area is vital to predict those who would most benefit from treatment (i.e. those with LTBI and a high risk of progression to ATB) and to identify those who have immunologic memory of disease but are not infected, and thus would not benefit from treatment.

To diagnose ATB, most of the world relies on established techniques such as sputum acid fast bacillus (AFB) staining and traditional culture techniques. These laborious and time-consuming assays are being replaced by more modern molecular tests. In particular, the GeneXpert nucleic acid-based test not only can detect Mtb in the sputum of patients with active disease but also can identify the presence of resistance to rifampicin, one of the essential first-line drugs for ATB treatment. Thus, where this test is available, patients with suspected ATB can receive a prompt molecular diagnosis and a more effective initial treatment for drug-resistant TB.

Antibiotics to treat TB have been available for over 50 years, but a major obstacle to successful therapy is that chemotherapy against TB requires long-term multidrug combinations and strict adherence to treatment. The current first-line chemotherapy for drug-susceptible TB involves a combination of rifampin (RIF), isoniazid (INH), pyrazinamide (PZA), and ethambutol (EMB) for the first 2 months, followed by 4 months of RIF and INH [22–24]. Mechanisms of action of these drugs have been elucidated, demonstrating complex roles of drug activation and binding to targets in Mtb [24]. TB can be cured over a 6-month period assuming the initial organism is drug susceptible and therapy is completed with adherence to a daily drug regimen. However, if these criteria are not met, i.e. if the initial organism is resistant to some of the drugs or if the patient is inconsistent with their therapy, cure can be significantly delayed. In fact, a significant threat to global TB control is the emergence of multidrug-resistant (MDR) and extensively drug-resistant (XDR) TB, as MDR/XDR-TB treatments are more complicated, often requiring longer treatment regimens with second-line agents [25]. MDR-TB is caused by Mtb strains resistant to INH/RIF and XDR-TB reflects resistance to INH/RIF plus any fluoroquinolone and at least one of the three second-line drugs, respectively [26]. Although MDR and XDR-TB primarily emerge by the development of mutations in drug targets such as *katG* and/or *inhA* (INH resistance), *rpoB* (RIF resistance), *pncA* (PZA), and *embCAB* operon (EMB resistance) [27–30], the host–pathogen dynamics underlying drug resistance are not fully understood, as mutations that confer antibiotic resistance in Mtb can evolve dynamically over time. Fortunately, owing to the recent development of several new classes of drugs against TB, therapeutic regimens for MDR and XDR-TB show promise [31]. Nevertheless, further studies are needed to better understand the relationship between the development of drug resistance and virulence, as well as to expand our knowledge of the molecular basis of Mtb pathogenesis, thereby contributing to new treatments for TB.

Evolutionary and comparative analyses of Mtb strains have identified virulence factors essential for Mtb survival and propagation inside the host, as well candidate genes for the development of new vaccines and anti-TB drugs. Studies using Mtb transposon mutant libraries and mouse model populations such as The Collaborative Cross and Diversity Outbred (inbred and outbred mouse lines, respectively) have identified a large number of bacterial and host genes, in some cases dependent on host genotype, that impact Mtb survival in vivo [32–34]. Mtb is currently divided into nine phylogeographically distinct lineages (L1-L9), widely distributed around the world [35–37]. Paleomicrobiology studies have suggested that the interaction between mycobacteria and humans began approximately 73,000 years ago and that TB was carried out of Africa through migrations during the Neolithic period [35,38]. The successful, long-standing association between Mtb and humans may have helped shape the biology and epidemiology of TB, as evolutionary “modern” lineages appear to be correlated with less severe early inflammatory responses, more efficient transmission rates, and differences in the emergence of MDR/XDR-TB [39–41].

The current review will summarize our knowledge of Mtb virulence factors, based on their molecular structures, host targets, and functions in pathogenesis. We have categorized them into two subgroups: non-protein and protein virulence factors. As the number of studies on the host–pathogen interactions in the context of Mtb is vast, it is outside the scope of this review to discuss all known virulence factors, and we regret omitting some topics due to space limitations. Before discussing the virulence factors themselves, we provide a brief overview of the life cycle of Mtb to offer broad context on their roles during infection. Our aim is to provide a framework to better understand the pathogenicity of one of the deadliest infectious agents in human history and highlight new potential targets for anti-TB therapy.

## Overview of infection

**Figure 1.**
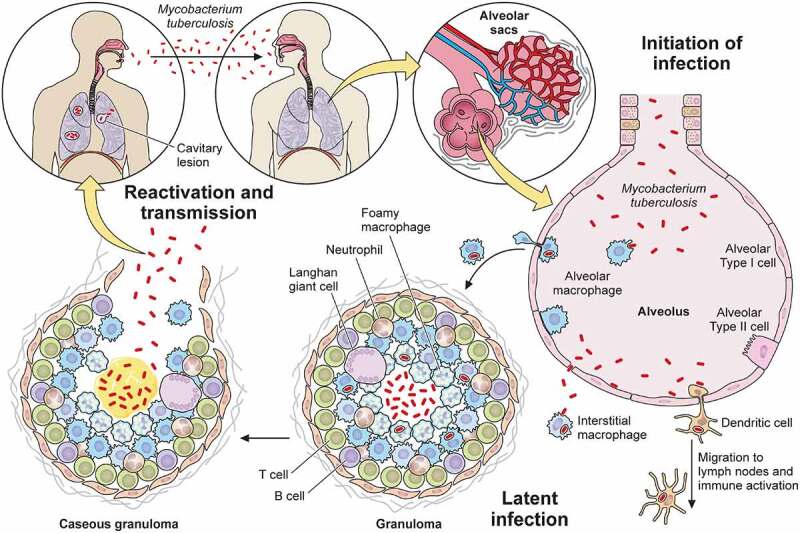
Overview of Mtb infection. Mtb enters the human body through the airway where it engages the innate immune system within the alveolar space. Macrophages and dendritic cells ingest the bacteria, recruiting new cells and activating adaptive immunity. Together, the innate and adaptive immune systems collaborate to eradicate the bacteria or restrict its active replication within a granuloma. Active tuberculosis occurs either after primary infection or after reactivation due to immunodeficiency, leading to symptomatic disease and transmission to a new host to start a new infection cycle.

Pulmonary TB is both the most common manifestation of TB and the mechanism of its transmission. Since Mtb can only survive and persist within living organisms – it does not appear to have an environmental niche [35] – we consider the Mtb life cycle initiated when it reaches the airway and lung (Figure 1). This first encounter between the airway and bacillus is also called primary infection.

Mtb first enters through the nose or mouth, encounters cells in the upper airway, and in most cases passes into the distal lung to arrive in the alveolar space. To survive and establish infection, Mtb then invades beyond the mucosal or alveolar epithelium. As it flows through the upper and lower airways, Mtb can infect epithelial cells [42] and microfold cells encountered in nasal associated mucosal tissue or bronchus-associated mucosal tissue [43,44] as an initial route to transit from the airway. Once the upper and larger distal airways are passed, the next site of interaction with the host occurs within the alveolus.

Because alveoli are continuously exposed to airborne particulates and pathogens, they also contain specialized innate immune cells known as alveolar macrophages that sample and engage with airborne antigens. In addition to alveolar macrophages, dendritic cells found within the interstitial space can also interact with airborne particles. Thus, during primary infection, Mtb that reaches the alveolus infects alveolar macrophages that reside in the alveolar space, as well as interstitial dendritic cells [42,45,46]. As an alternative route of entry, type II alveolar epithelial cells can also be infected by Mtb [47,48]. These cells fail to control infection with associated high rates of cell death [49]. Since alveolar epithelial cells vastly outnumber alveolar macrophages, this route may represent an important mechanism through which Mtb traverses the mucosa.

Mtb-infected alveolar macrophages and dendritic cells both serve as early reservoirs of infection and function to activate an adaptive immune response. Infected alveolar macrophages migrate from the alveolar sac into the interstitial space [1]. In some circumstances, the infected macrophages will then reside within the interstitium [50], and in other circumstances, infected macrophages in addition to infected dendritic cells migrate from the lung to draining lymph nodes to prime and activate T and B cells that function to limit progression of infection [51–53]. Within the interstitium, resident interstitial macrophages engulf extracellular bacteria that escape initial phagocytosis or after their release from dying cells. Both types of infected macrophages – alveolar and interstitial – along with non-infected macrophages, inflammatory monocytes, neutrophils, and T cells recruited by the inflammation and tissue damage then form the characteristic TB granuloma. Whether this multicellular structure exists to limit or enhance Mtb infection is still unclear [54,55]. However, in most primary infections, infection is controlled, either through complete eradication of the bacteria leaving behind only immunologic memory of the interaction or through formation of a stable granuloma.

From the host’s perspective, a well-formed and stable granuloma limits the progression of infection and constrains any tissue damage to a small, well-circumscribed region. Most Mtb-infected individuals will contain the disease at this step and be asymptomatic. From the bacterial perspective, the granuloma permits the bacteria to maintain a state of dormancy and thus avoid clearance by the immune system [56]. Thus, contained, Mtb may persist indefinitely. Aside from during autopsy or surgery where bacilli can be cultured from surreptitiously identified granuloma, this condition can currently only be diagnosed with TST or IGRA. Recently, some have questioned whether the vast numbers of individuals with LTBI, ~1/4 to 1/3 of the world’s population as estimated by the WHO, truly co-exist with viable organisms versus having cleared the infection and demonstrating an appropriate amnestic cell mediated immune response [57,58].

Notwithstanding the question of prevalence of “true” LTBI, some infected individuals proceed to active infection, either directly after the primary infection or after “reactivation” from latent infection, generally in the setting of immunosuppression [59]. Often when this occurs, the structure of the granuloma changes and is associated with the presence of a material known as caseum which can facilitate more rapid Mtb growth. The central region of caseum can liquify, creating an even more favorable growth environment. Mtb can then spread beyond the lung to other parts of the body either via lymphatics [60] or blood vessels [61]. Ultimately, granuloma liquefaction, which is also associated with tissue destruction [62], can facilitate the spread of tuberculosis to naive hosts through airborne transmission [63,64].

In the absence of treatment, ATB has a high mortality rate [65]. During this phase, Mtb stimulates airway nociceptive neurons to produce a chronic, bloody cough that is one of the most characteristic symptoms of ATB disease. This persistent cough is also one vector of transmission that allows Mtb to escape and propagate infection [63]. In this way, Mtb may renew its life cycle in a naive host.

## Virulence factors

Considering the ability of Mtb to infect a variety of mammalian hosts [66], manifest clinically in humans as both latent and active infection, and dwell in the air within motes of airborne particles to be transmitted to naive hosts, it comes as no surprise that it has evolved a variety of approaches to establish infection and survive within a variety of cells, tissues, and environments. Below we discuss the impact of these virulence traits, distinguishing between non-protein virulence factors such as lipids and sugars, and canonical proteinaceous virulence factors.

## Non-protein virulence factors

Though classically protein virulence factors have been the focus of microbiologists, organisms have evolved to leverage many other cellular components to facilitate their growth and survival within replicative niches. In this section, we discuss the functions of non-proteinaceous virulence factors organized by molecule class including lipids/glycolipids, glycans, nucleic acids, and metabolites. Many of these molecules are key components of the cell surface, and interact with host targets after secretion, shedding, or while attached to the bacterial surface. Indeed, mycobacterial and related species have a unique cell envelope with several components playing a role in virulence [67,68]. In addition to impacting the host through direct interactions, the structural rigidity of the mycobacterial cell envelope provides protection in the form of greater impermeability to antimicrobial molecules produced by the immune system the bacteria encounter in infection. Furthermore, the importance of the mycobacterial cell envelope to its survival in the host is highlighted by the fact that two of the first-line antibiotics to treat TB, isoniazid and ethambutol, target the biosynthesis of mycolic acids and arabinogalactan, arabinomannan, and lipoarabinomannan, respectively. The biosynthesis of cell surface molecules is discussed in great detail elsewhere [69,70], while here we focus on their roles in the context of host interactions [69–72].

### Lipids and glycolipids

Many bacteria are known to shed components of their outer membrane or cell wall, oftentimes having the capacity to dramatically impact host biology. The best studied of these, the lipopolysaccharide (LPS) of gram-negative bacteria, mediates a vast array of host responses through its binding to pattern recognition receptors such as toll-like receptor 4 (TLR4) on the cell surface and caspase 4/11 in the cytoplasm [73]. In a similar vein, mycobacterial lipid and glycan molecules can either be shed or potentially transported directly into the host via secretion or membrane vesicles [74–76]. As noted above, such molecules may also serve to buttress the mycobacterial cell wall and/or create impenetrable barriers to host defences [77]. Although these activities are not mutually exclusive, below we focus on the virulence functions of these complex molecules (Table 1).

**Table 1.** Non-protein virulence factors of Mtb.

Molecule	Abbreviation	Category	Function	References
Phthiocerol Dimycocerosates	PDIMs	Lipid	Masks PAMPs, induces membrane rupture	[78]
Mycolic acid	MA	Lipid	TLR2 inhibitor, induces MCP-1	[79–81]
1-tuberculosinyladenosine	1-TbAd	Lipid	Lysosomal escape, inhibits acidification	[82–84]
Phenolic glycolipid	PGL	Glycolipid	Inhibits cytokine activity	[85–88]
Sulfolipid-1	SL-1	Glycolipid	Cough induction	[63,89]
Trehalose dimycolic acid	TDM	Glycolipid	Granuloma formation, induces TNF-α, IL-6, MCP-1 cytokines	[90–95]
Trehalose monomycolic acid	TMM	Glycolipid	Induces TNF-α, IL-6, MCP-1, other cytokines	[96–98]
Lipomannan	LM	Glycolipid	TLR2/TLR4 agonist, induces IL-12 production	[99,100]
Mannosylated Lipoarabinomannan	ManLAM	Glycolipid	Phagocytosis & phagosome maturation arrest	[101–104]
Phosphatidyl inositol mannosides	PIMs	Glycolipid	TLR2 & C-type lectin agonist	[105–109]
Diacyl trehalose, pentaacyl trehalose	DAT, PAT	Glycolipid	Inhibits IL-12 production, induces IL-10 & TNF-α, inhibits NO production	[110–113]
Arabinomannan	AM	Glycan	Similar to LAM	[114–118]
Mannan		Glycan	Similar to LM	[114–118]
α-glucan		Glycan	Induces IL-10 production, inhibits CD1 expression, induces phagocytosis	[119–121]
Deoxyribonucleic acid	DNA	Nucleic acid	Induces IFN-β, triggers autophagy	[122–125]
Ribonucleic acid	RNA	Nucleic acid	Induces IFN-β	[126]
Metabolites		Small molecule metabolites	Metabolites impact Mtb pathogenesis	[127–133]

### Mycolic acid

**Figure 2.**
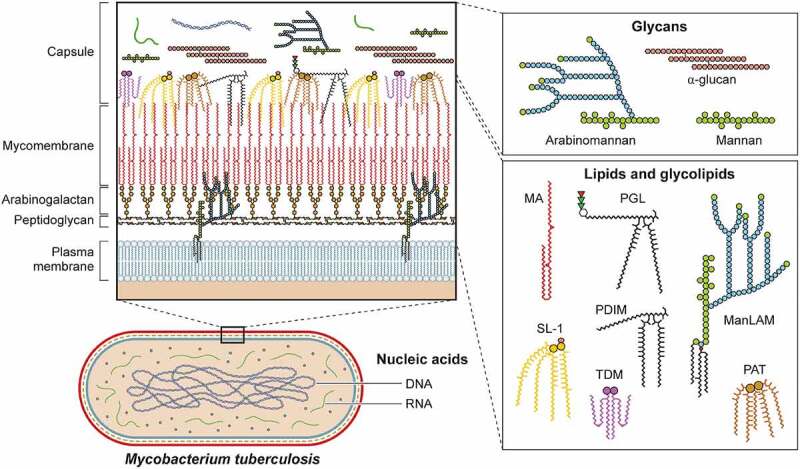
Non-protein virulence factors of Mtb. Schematic of Mtb lipids, glycolipids, glycans, and nucleic acids that contribute to virulence. TDM: trehalose dimycolate, SL-1: sulfolipid-1, PGL: phenolic glycolipid, MA: mycolic acid, PAT: pentaacyl trehalose, PDIM: phthiocerol dimycocerosates, ManLAM: mannosylated lipoarabinomannan.

Mycolic acids, long chain fatty acids ranging between (C60-C90), are the primary constituents of the inner leaflet of the outer membrane. Mycolic acids are covalently linked to the arabinogalactan layer or are present as free mycolic acid within the capsule (Figure 2) [134,135]. This region is also known as the mycomembrane for the abundance of this lipid species. In alveolar epithelial cells, mycolic acids inhibit TLR2 leading to a decrease in IL-8 production [79]. Free mycolic acids can activate the DAP12-associated triggering receptor expressed on myeloid cells 2 (TREM2) on innate immune cells, which in turn increases MCP-1 production and recruitment of Mtb permissive macrophages [80]. Overproduction of MCP-1 correlates with susceptibility to ATB [136]. Thus, regulation of inflammation via IL-8 and MCP-1 is but one of several mechanisms by which mycolic acids influence host immunity [137]. That Mtb can control both the abundance and structure of mycolic acids in response to its environment [81] suggests that temporal and spatial control of mycolic acids allows Mtb to fine-tune the host response during various stages of infection.

### Trehalose monomycolate and trehalose dimycolate

Trehalose monomycolate (TMM) and trehalose dimycolate (TDM) are abundant glycolipids formed by the conjugation of mycolic acids to the disaccharide trehalose via ester bonds [138,139]. TMM is a precursor to mycolic acid and presents within the cell envelope where it can be shed or secreted by Mtb by an unknown mechanism [75]. TDM is also known as cord factor for mediating the formation of long filaments or “cords” when Mtb is extracellular. Because the interaction of TMM is similar to TDM, here we focus on the virulence capacity of TDM, with the recognition that TMM has overlapping activities. Monocyte-Inducible C-type Lectin (Mincle), a pattern recognition receptor (PRR) present in macrophages and dendritic cells, binds to the trehalose motif of TDM [96,140]. This interaction triggers SH2-domain-containing inositol polyphosphate 5’ phosphatase 1 (SHP-1) and Fc gamma receptor IIB (FcγRIIB) leading to inhibition of phagosome arrest [90–92]. Mincle also activates the PI3K-AKT-GSK3 signaling pathway leading to the production of TNF-α, IL-6, and MCP-1 with subsequent recruitment of monocytes and neutrophils [141–143]. Notably, by modifying TDM via enzymatic cyclopropanation, Mtb can temporally modulate the host immune response during infection [144,145].

### Phthiocerol dimycocerosates

Another major class of lipids with diverse immunoregulatory activities are the phthiocerol dimycocerosates (PDIM) [78]. Briefly, these lipid species are present on the outer leaflet of the outer membrane and may be secreted or shed from mycobacteria in a similar manner as described previously. While several glycolipids are known to interact with host factors (Table 1), PDIM is thought to mask pathogen-associated molecular patterns (PAMPs) of Mtb, allowing the bacteria to evade recognition by TLRs [146,147]. After phagocytosis, PDIM is suggested to have a role in membrane rupture including both phagosome and mitochondria membranes [148,149]. Though damaged phagosomes signal the host cell to initiate degradative autophagy, a cell-intrinsic host defence, PDIM also inhibits MyD88 signaling, leading to a decrease in autophagy [150,151].

### Phenolic glycolipids

Phenolic Glycolipid (PGL), the covalent linkage of PDIM to a phenolic moiety and one to 4 sugars, is another important component of the mycobacterial outer membrane [152]. While some epidemiologic evidence links PGL production to more “hypervirulent” lineage 2 strains of Mtb [85–87] owing to a disruption of polyketide synthase gene pks15/1 essential for PGL production in lineage 4 Mtb strains [153,154], recent work has identified “ancient” or “ancestral” subspecies within lineage 2 strains that also contain mutations in pks15/1 [155]. PGL activates the cytosolic surveillance pathway and induces MCP-1 secretion (also known as CCL2) [156,157]. CCL2 in turn interacts with host chemokine receptor 2 (CCR2) and recruits Mtb permissive macrophages [146]. In support of this model, a human genetic polymorphism that increases MCP-1 production is associated with increased likelihood of developing pulmonary tuberculosis [136]. However, CCR2 knockout mice are not more susceptible to infection by a lineage 4 Mtb strain, which genetically lacks PGL, and while CCR2 knockout mice do display enhanced susceptibility to a lineage 2 strain (HN878), this outcome is PGL independent [157]. PGL may also modulate the immune system by antagonizing the function of TLR2 [158], or by inhibiting inflammatory cytokine activity by interfering with T-cell receptor signaling [88].

### Polyacyl trehaloses

The disaccharide trehalose is a precursor for multiple Mtb glycolipids. In addition to the covalent attachment of mycolic acids as discussed above, trehalose can be acylated in a variety of positions [159]. Here, we concentrate on the role of diacyl trehalose (DAT), because of its greater implication in Mtb virulence. When dendritic cells are exposed to DAT, production of pro-inflammatory interleukin-12 (IL-12) is downregulated, and production of the anti-inflammatory cytokine IL-10 is increased [160]. In the context of macrophage infection with Mtb, both DAT and triacyl trehalose (TAT) downregulate expression of inducible nitric oxide synthase (iNOS), leading to reduced nitric oxide (NO) production [161]. Like TDM, acyl trehaloses bind cell surface Mincle receptors, triggering a signaling cascade that increases production of TNF-α and other cytokines [110,111]. In parallel, DAT may mask the interaction of PRRs with other lipid virulence factors [162,163]. DAT may also impact adaptive immune responses to Mtb as it inhibits T cell proliferation and cytokine production through protein kinase C (PKC) activation and inhibition of mitogen-activated protein kinase (MAPK) [164,165]. Because DAT, TDM, and other lipids are presented by the MHC class I CD1 protein to initiate T-cell activation [110], the ability of DAT to inhibit T cell activities may prevent a more robust anti-Mtb adaptive immune response.

### Sulfolipids

While sharing structural similarity to tetra acyl trehalose, sulfolipid-1 (SL-1) has a sulphate group at the 2’ carbon of trehalose (Figure 2). SL-1 is one of the most abundant lipids in Mtb and represents approximately 1% of its dry weight [166]. SL-1 does not have a classic virulence function, as Mtb strains lacking SL-1 are not attenuated in mice or guinea pigs in traditional infection models [167,168]. However, recent work has shown that SL-1 is sufficient to activate nociceptive neurons from mice and humans and to stimulate a cough reflex in guinea pigs [63]. SL-1 production is reduced during latent infection and upregulated during active infection [169]. Taken together, Mtb may regulate SL-1 production to facilitate another critical stage of infection, namely, transmission.

### Phosphatidylinositol mannosides/Lipomannan/Mannosylated lipoarabinomannan

The most abundant glycolipids present in the mycobacterial cell envelope are phosphatidyl-myo-inositol mannosides (PIMs) [134,170–172]. Acyl phosphatidyl-myo-inositol dimannoside (AcPIM2) comprises most of the inner membrane in most mycobacteria species [134]. Furthermore, AcPIM6, one of the other major PIM species [173,174], helps maintain cell envelope integrity [175]. The structure of lipomannan (LM) represents the decoration of PIM species with multiple mannose sugars [174]. Finally, lipoarabinomannan (LAM) is formed by the attachment of multiple arabinose residues to LM, and when LAM is capped by another mannan group, forms a structure known as ManLAM (Figure 2). Through its engagement of the scavenger receptor, CD36, ManLAM inhibits nitric oxide production [176]. Recognized by their mannose residues, PIMs, LM, LAM, and ManLAM interact with several C-type lectin receptors including mannose receptor (MR), dendritic cell immunoactivating receptor (DCAR), and dendritic cell-specific intercellular adhesion molecule-3-grabbing non-integrin (DC-SIGN, also known as CD209) [105,177]. C-type lectin receptors initiate phagocytosis in macrophages, dendritic cells, and neutrophils [178], and these interactions may facilitate mycobacterial uptake by alveolar macrophages to provide an early replicative niche. In its most well-characterized activity, ManLAM protects Mtb from degradation in the phagolysosome of macrophages by arresting the maturation and acidification of phagosomes [179–182] through both inhibition of phosphoinositide-3-kinase [182,183] and its interaction with lipid rafts leading to delayed phagosome-lysosome fusion [184]. Thus, these phosphatidylinositol containing glycolipids can not only engage host receptors to facilitate Mtb uptake into host cells but also prevent their degradation by cell intrinsic immune mechanisms.

### Glycans

Peptidoglycan and arabinogalactan within the cell envelope play a critical role for Mtb infection and the overall impermeability of its cell envelope [71,185,186]. The glycans found within the 40 nm capsular space (Figure 2), namely mannan, arabinomannan, and α-glucan [101,119,187–190], play important roles in Mtb pathogenesis. It should be noted that when investigators grow and prepare Mtb for animal infection experiments, to prevent bacterial clumping, Mtb is often grown in Tween-80, which acts as both a nutrient and surfactant. However, Tween-80 also disrupts the Mtb capsule, thus making interpretation of experiments that characterize the role of the Mtb capsule in initial infection challenging [187,191–194]. Mannan and arabinomannan share structural similarities to LM and LAM, respectively. These glycans similarly manipulate the host, and since they form a part of the bacterial capsule, they are more likely to interact with host cells prior to LAM and LM [114–117,195]. α-Glucan is the major capsular glycan identified during most experimental conditions [189,196]. Highlighting its role in virulence, an α-glucan deficient Mtb strain is highly attenuated in a mouse model of infection [119] possibly owing to its inability to modulate innate and adaptive immunity [120,197].

### Nucleic acids

Through its specialized Type VII secretion system called ESX-1, Mtb can rupture or damage the phagosome and enter the cytosol [198]. Once that occurs, mycobacterial DNA has been proposed to enter the cytosol to interact with cyclic GMP-AMP synthase (cGAS) [122–125] though other studies have suggested that host mitochondrial DNA also acts as a cGAS ligand [199]. Subsequent activation of STING via cGAS-derived cGAMP results in downstream signaling that leads to robust production of interferon-beta (IFN-β), which is detrimental to the host in the context of Mtb infection [200,201], potentially through decreased macrophage metabolism [202]. In parallel, Mtb secretes RNA into the host cytoplasm to induce IFN-β via the host RNA sensing pathway mediated by RIG-I and MAVS [126]. While Mtb may benefit from inducing excess IFN-β, activation of STING also induces autophagy, a host defence that can facilitate bacterial clearance [203].

## Protein virulence factors

**Figure 3.**
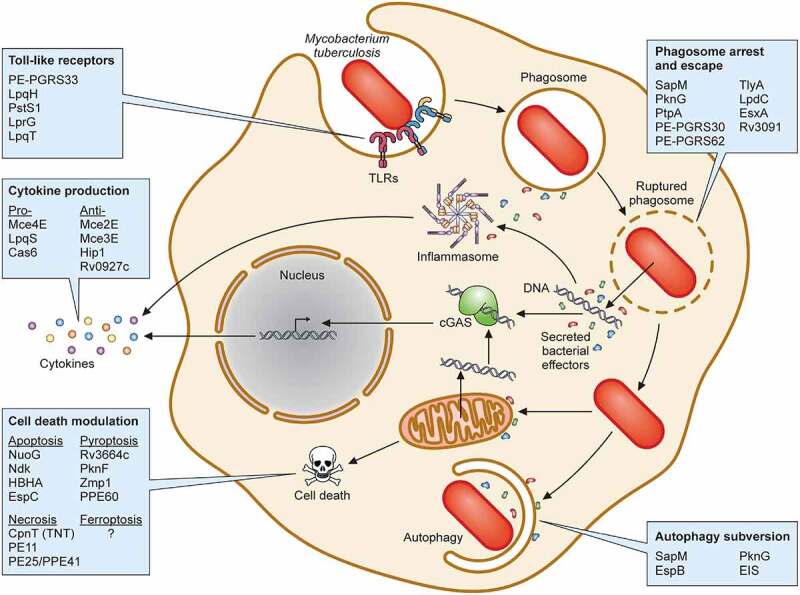
Protein virulence factors of Mtb. Schematic representation of Mtb interactions within infected macrophages highlighting (i) toll-like receptors, (ii) phagosome arrest and escape, (iii) autophagy subversion, (iv) cell death modulation, and (v) cytokine production. Each box contains representative Mtb virulence factors that engage host cell biologic processes detailed in the text.

Since Mtb is an obligate intracellular pathogen, it comes as no surprise that it has dedicated a significant proportion of its proteome to proteins that enhance its survival. In this section, we focus on several categories of Mtb protein virulence factors that directly impact the host, taking a predominantly bacterial-centered view. We group virulence determinants into the following categories based on their functions in the context of their interaction with the host: (i) modifiers of TLR2 activity, (ii) regulators of cytokine production, (iii) disruptors of phagosome function, and modulators of (iv) autophagy, and (v) cell death. Wherever possible, we highlight virulence protein activities (Figure 3) but also provide a summary table of proteins with known virulence function (Table 2).

**Table 2.** Protein virulence factors of Mtb.

Group	Protein	Rv number	Category	Function	Refs
Modulation of TLR2 signaling	LpqH	Rv3763	Lipoprotein	TLR2 agonist, induces T cell proliferation	[204,205]
	PE_PGRS33	Rv1818c	PE/PPE family member	Participates in Mtb cell entrance via TLR2, induces TNF-α release, induces apoptosis, induces mitochondrial fusion	[206–208]
	LprG	Rv1411c	Lipoprotein	TLR2 agonist, translocates glycolipids to cell surface, induces mitochondrial fission	[209,210]
	PstS1 phoS1	Rv0934	Lipoprotein	TLR2 and TLR4 agonist	[211]
	CFP 32	Rv0577	Putative glyoxylase	TLR2 agonist	[212–214]
	LpqT	Rv1016c	Lipoprotein	TLR2 agonist, inhibits MHC II	[215–217]
	PE-PGRS61	Rv3653	PE/PPE family member	TLR2 agonist	[218,219]
	PPE34	Rv1917c	PE/PPE family member	TLR2 agonist, induces DCs maturation	[220]
	PPE26	Rv1789	PE/PPE family member	TLR2 agonist, induces TNF-α, IL-6, and IL-12 p40 secretion, induces necrosis	[221,222]
	PPE57	Rv3425	PE/PPE family member	TLR2 agonist	[223]
	PPE60	Rv3478	PE/PPE family member	TLR2 agonist, induces DCs maturation, induces pyroptosis	[224,225]
	PE-PGRS11	Rv0754	PE/PPE family member	Interacts with TLR2, induces DCs maturation, required for oxidative stress	[226,227]
	PE-PGRS17	Rv0978c	PE/PPE family member	Interacts with TLR2, induces DCs maturation	[226]
Modulation of cytokine production	Mce2E LprL	Rv0593	Lipoprotein	Suppresses host immune response, epithelial cell proliferation	[228]
	Mce3E LprM	Rv1970	Lipoprotein	Down-regulates TNF and IL-6 expression	[229]
	Mce4E LprN	Rv3495c	Lipoprotein	Induces T-cell proliferation and increases production of TNF-α and IFN-γ cytokines	[230,231]
	RpfB	Rv1009	-	Induces T-cell proliferation, is required to Mtb reactivation,	[232,233]
	LpqS	Rv0847	Lipoprotein	Induces TNF-α, IL-12, and IL-6 secretion	[234]
	EspL	Rv3880c	ESX1 secretion system	Induces IL-1β, IFN-β, ISG15, and IL-6 production, stabilizes EspE, EspF and EspH	[235]
	-	Rv1987	RD2 gene	Induces TH2 immune response	[236,237]
	PE27	Rv2769c	PE/PPE family member	Induces Th1 response polarization	[238]
	Cas6	Rv2824c	Crispr/cas	Induces apoptosis, Upregulates IL-6, IL-1β and TNF-α	[239,240]
	Hip1 CaeA	Rv2224c	Protease	Reduces proinflammatory response, hampers DCs maturation, and inhibits antigen presentation and T cell responses	[241–243]
Phagosome arrest & autophagy subversion	SapM	Rv3310	Acid phosphatase	Suppresses phagosome maturation and autophagy flux	[244–247]
	ESAT-6 EsxA	Rv3875	ESX-1 secretion system	Diverse roles including phagosome rupture and translocation to the cytosol	[248,249]
	PE_PGRS30	Rv1651c	PE/PPE family member	Inhibits phagosome-lysosome fusion, reduces IL-12, TNF-α, and IL-6, required for lung tissue colonization	[250,251]
	SecA2	Rv1821	Secretion system	Protein export system that participates in the phagosome and autophagosome maturation arrest	[247,252]
	PtpA MptpA	Rv2234	Hydrolase, Protein phosphatase	Inhibits phagosome-lysosome fusion, phagosome acidification, suppresses innate immune response	[253–255]
	PknG	Rv0410c	Protein Kinase	Inhibits phagosome-lysosome fusion, inhibits autophagy flux, suppresses innate immune response, latent Mtb reactivation	[256–258]
	MPT63	Rv1926c	-	Forms membrane pores	[259,260]
	PE_PGRS62	Rv3812	PE/PPE family member	Inhibits phagosome-lysosome maturation, induces ER stress-mediated apoptosis	[261,262]
	TlyA	Rv1694	RNA methyl-transferase	Inhibits phagosome maturation, increases IL-12 secretion, reduces IL-1β and IL-10 secretion	[263,264]
	LpdC	Rv0462	Oxidoreductase	Inhibits phagosome maturation, induces DCs maturation	[265,266]
	Ndk	Rv2445c	Kinase, Transferase	Inhibits phagosome maturation	[267]
	-	Rv3091	Patatin-like phospholipase	Phagosome escape	[268]
	EsxH	Rv0288	ESX-1 secretion system	Inhibits phagosome maturation	[269]
	EIS	Rv2416c	Acyltransferase	Inhibits autophagy, suppress TNF-α and IL-6 secretion and ROS generation	[270–272]
	EspB	Rv3881c	ESX-1 secretion system	Inhibits autophagy	[273]
Cell death modulation	HbhA	Rv0475	Cell wall protein	Induces apoptosis, essential for the infection of lung epithelial cells and extrapulmonary infection	[274–277]
	EspC	Rv3615c	ESX-1 secretion system	Induces ER stress-mediated apoptosis	[278]
	NuoG	Rv3151	Translocase	Inhibits apoptosis	[279,280]
	-	Rv3654c	Secreted protein	Inhibits apoptosis	[281]
	-	Rv3655c	Secreted protein	Inhibits apoptosis	[281]
	PknE	Rv1743	Protein Kinase	Inhibits apoptosis, induces TNF-α and IL-6 secretion	[282,283]
	-	Rv3033	-	Inhibits intrinsic apoptosis	[284]
	CpnT	Rv3903c	Hydrolase Porin	Induces necrotic cell death	[285,286]
	PE25 PPE41	Rv2431c/Rv2430c	PE/PPE family member	Induces necrosis, induces DCs maturation	[287]
	PE11 LipX	Rv1169c	PE/PPE family member	Induces necrotic cell death, reduces IL-6 production, alters cell wall lipid composition	[288–290]
	Zmp1	Rv0198c	Metallo-protease	Induces necroptosis, inhibits phagosome-lysosome fusion, induces TNF-α, IL-6, IL-1β, MCP-1, MIP-1β, and IL-8 secretion	[291,292]
	-	Rv3364c	-	Inhibits pyroptosis	[293]
	PknF	Rv1746	Protein kinase	Inhibits pyroptosis	[294]
Other functions	LprI	Rv1541c	Lipoprotein	Lysozyme inhibitor	[295]
	CFP-10 EsxB	Rv3874	ESX-1 secretion system	EsxA chaperone, activates neutrophils	[296,297]
	Acr1	Rv2031c	Latency-associated protein	Inhibits maturation and differentiation of dendritic cells and macrophages	[298,299]
	-	Rv0431	Glycoprotein	Regulates vesiculogensis	[300–302]
	GroEL2	Rv0440	Chaperonin	Modulates DC responses	[303]
	-	Rv3194c	Serine protease	Degrades components of complement	[304]
	MPT64	Rv1980c	-	Up-regulates bcl-2, modulates ER stress	[305,306]
	PknH	Rv1266c	Protein kinase	Regulates LAM and AG	[307–309]
	RipA	Rv1477	Hydrolase Protease	Endopeptidase, activates TLR4	[310]
	PPE38	Rv2352c	PE/PPE family member	Coordinates ESX-5 effector secretion, regulates NF-kB activity	[311,312]

### TLR2 activity modifiers

A myriad of host cell receptors can recognize Mtb cell wall ligands, including scavenger receptors, C-type lectin receptors, and TLRs [313]. Among the TLRs, TLR2 plays a major role in detecting Mtb [314,315]. After TLR2 activation, the adapter protein myeloid differentiation primary response protein 88 (MyD88) is recruited and an intracellular signaling cascade is triggered [316,317]. Ultimately, this signaling pathway leads to the production of pro-inflammatory cytokines such as TNF-α, IL-1β, and IL-12, chemokines, and antimicrobial molecules (reactive oxygen intermediates (ROI) and reactive nitrogen intermediates (RNI)), which all play a role in controlling Mtb infection [318,319].

Considering its importance to the host, Mtb has developed multiple mechanisms to exploit TLR2 activation for its benefit. One mechanism involves leveraging the PE and PPE protein families, which are characterized by a conserved Pro-Glu and Pro-Pro-Glu motifs in their N-terminal region, respectively [318]. Almost 10% of the Mtb genome is devoted to PE/PPE proteins [320] and many members of these families interact with TLR2 to modulate the host innate immune response including PPE34, PE-PGRS11, PE-PGRS17, PE-PGRS33, PPE26, PPE57, and PPE60 [220,221,223,224,226,314]. The best characterized of these is PE-PGRS33, which interacts with TLR2 in a Ca^2+^-dependent manner [206,218] resulting in the secretion of pro-inflammatory cytokines and chemokines [207,321].

In addition to PE/PPE proteins, other Mtb virulence factors have been described as modulators of TLR2 signaling. Lipoproteins are both well-established TLR2 ligands and abundant components of the Mtb cell envelope. To that end, there are multiple Mtb lipoproteins that may engage and modulate TLR2 including LprG [209], LpqH [204], LpqT [215] and PstS1 [211]. Of note, the context of interaction may play an important role in the outcome of the response, such that free protein may cause a different response in the host than bacterial associated protein. Thus, when recombinant LprG [209], LpqH [204], and LpqT [215] are added to macrophages, they activate pro-inflammatory cytokine secretion, inhibit antigen processing and IFN-γ-mediated MHC-II antigen presentation to T cells in a TLR-dependent manner. Likewise, PstS1 induces TNFα and IL-6 in primary human monocytes predominantly through TLR2 activation [220]. However, experiments conducted using *M. smegmatis* expressing Mtb LpqT [216] or LpqH [322] yield conflicting results to those using purified proteins. For example, neither *M. smegmatis* expressing LpqT nor LpqH suppress macrophage MHC-II expression [216,322]. Both studies also showed that *M. smegmatis* expressing LpqT or LpqH decrease pro-inflammatory cytokine secretion [216,322], suggesting that they may act to dampen TLR2 activity. Taken together, it remains unclear how these and other Mtb lipoproteins impact TLR2 activity, and whether direct bacterial contact with innate immune cells impacts the TLR2 response. Indeed, it was demonstrated recently that phagosome membrane damage mediated by PDIM and ESX-1 are necessary to interfere with the host TLR2 response [323], indicating that cytoplasmic access may be a critical requirement to impact the TLR2 signaling system.

### Regulators of cytokine production

While modulating TLR signaling is one approach that Mtb takes to impact the host response and later cytokine production, Mtb uses other effector proteins to manipulate signaling pathways important for appropriately timed and regulated cytokine production. By inducing anti-inflammatory cytokines or suppressing pro-inflammatory cytokines, Mtb can fine-tune the host innate immune response to enhance its virulence.

The mammalian cell entry (*Mce*) operons are critical both for initial phagocytosis and manipulation of host cell cytokine responses [230,324–326]. Within these operons, several Mce lipoproteins are known to manipulate cytokine production via inhibition of phosphorylation cascades. For instance, Mce2E, also known as LprL, inhibits the activation of ERK and JNK MAPK signaling pathways and K48 ubiquitination of eEF1A1. Mce2E has a MAPK-docking motif that directly binds to mitogen-activated protein kinase 1 (MAPK1 or ERK2) and mitogen-activated protein kinase 8 (MAPK8 or JNK1), inhibiting their activation and resulting in decreased production of TNF-α and IL-6 [228]. Similarly, another Mce (Mce3E or LprM) binds to MAPK1, inhibiting its phosphorylation and thus reducing production of TNF-α and IL-6 [229]. Thus, a variety of Mce proteins function to suppress innate immune signaling pathways.

In addition to members of the Mce families, other Mtb virulence proteins modulate cytokine production. The serine hydrolase, Hip1, inhibits the production of several pro-inflammatory cytokines such as IL-12, IL-6, and TNF-α in dendritic cells [241]. Likewise, Rv0927c, a short-chain dehydrogenase/reductase (Rv0927c) also downregulates TNF-α, IL-1β, and IL-6 production, by preventing NF-kB activation [300,327]. In contrast, another lipoprotein, LpqS stimulates TNF-α and IL-12 production [234]. Recently, in an unexpected discovery, an Mtb Type III CRISPR/Cas system protein, Cas6, was demonstrated not only to be important for virulence of Mtb in a mouse model of disease but also a secreted virulence factor that induces IL-6, IL-1β, and TNF-α production via an unknown mechanism [239]. This study, among others, indicates that CRISPR/Cas is not only a bacterial defence against bacteriophages but can also function as a virulence factor. Overall, that a wide array of Mtb virulence factors can impact host cytokine production, and in some cases have opposing effects on the same cytokine (Table 2), suggests that fine-tuned control of both pro- and anti- inflammatory cytokines at various stages of infection is critical for Mtb virulence.

### Phagosome maturation arrest and phagosome escape

After recognition of Mtb ligands by host cell receptors on professional phagocytes, bacteria are internalized by phagocytosis and subsequently located within early phagosomes. During their maturation process, phagosomes acidify to pH ≤5 and fuse with lysosomes, forming phagolysosomes. In the absence of any intervention, the fate of these internalized organisms is killing by host antimicrobial processes and digestion by phagolysosomal hydrolases. However, Mtb has evolved sophisticated strategies to avoid the degradative phagolysosomal environment by delaying or inhibiting phagosome-lysosome maturation, and by mediating phagosome rupture and escape into the cytosol.

Several Mtb proteins are involved in arresting phagosome maturation. First identified in 2000, Mtb SapM is a phosphatase involved in phagosome-lysosome inhibition [244]. SapM dephosphorylates phosphatidylinositol 3-phosphate (PI3P) present in the phagosome membrane leading to inhibition of phagosome-lysosome fusion in the murine macrophage cell line RAW 264.7 [245] and PMA-differentiated human THP-1 macrophages (hereafter named THP-1 cells) [246]. The secreted protein PknG also inhibits phagosome-lysosome fusion favoring bacterial survival inside THP-1 cells through its interaction with GDP-bound Rab7L1 (Rab29 in mouse) on the Golgi complex, blocking its transition to active Rab7L1-GTP and, consequently, inhibiting Rab7L1-GTP recruitment to phagosomes [256]. Inhibition of Rab7L1-GTP recruitment to phagosomes blocks the maturation of phagosomes as evidenced by impaired recruitment of other phagolysosomal markers like early endosome autoantigen 1 (EEA1), Rab7, and LAMP2 [256]. PknG has also been implicated in host immunity impairment [257], LTBI reactivation [328], and autophagic flux inhibition [258]. Of note, both SapM and PknG are exported by the SecA2 protein export system [247] and then likely enter the cytoplasm through EsxA mediated phagosome disruption. Another Mtb phosphatase that interferes with phagosome maturation is PtpA, which blocks phagosome-lysosome fusion via dephosphorylation of VPS33B, a host protein involved in intracellular vesicle trafficking [253]. PtpA also binds subunit H of the macrophage vacuolar-H^+^ATPase (V-ATPase) protein complex, inhibiting phagosome acidification [253,329,330]. Thus, targeting of V-ATPase by PtpA hampers the proper function of several host proteins thereby inhibiting phagosome acidification and ultimately favoring Mtb infection in THP-1 macrophages [253]. PtpA activity is regulated by protein tyrosine kinase A (PtkA) and protein kinase A (PknA) through phosphorylation [331]. Like PknG, PtpA impacts other host cell pathways, including (i) modulating the host immune response by co-opting host ubiquitin [254], (ii) regulating expression of host genes [332], and (iii) blocking host cell apoptosis via GSK3 dephosphorylation [333]. Thus, Mtb secreted virulence factors with enzymatic activities like SapM, PknG and PtpA can be multifunctional.

In this vein, considering their high prevalence in the Mtb genome, among the multiple functions of PE/PPE proteins in host cells, they also participate in arresting the maturation of Mtb-containing phagosomes [250]. For example, during macrophage infection, an Mtb mutant lacking PE-PGRS30 demonstrated increased colocalization with LAMP-1 compared to wild-type Mtb, suggesting that PE-PGRS30 inhibits phagosome-lysosome fusion [250]. This mutant was also attenuated in a mouse infection model, highlighting its importance to Mtb virulence. Furthermore, when J774 macrophages are infected with *M. smegmatis* expressing Mtb PE-PGRS62, phagosome maturation is blocked through inhibition of Rab7 and LAMP-1 recruitment [261].

Other Mtb proteins involved in phagosome maturation arrest are TlyA and LpdC. TlyA impairs phagosome maturation by reducing recruitment of Rab5, Rab7, and EEA1 to the phagosome [334]. Rather than block recruitment of Rab proteins (a common feature of virulence factors), Mtb LpdC leads to retention of coronin-1 on the phagosome membrane [265]. Coronin-1 activates the Ca^2+^-dependent phosphatase calcineurin and calcineurin activity blocks fusion of phagosomes with lysosomes [335], suggesting a mechanism of action of LpdC in phagosome maturation arrest via coronin-1 and calcineurin.

Another strategy to subvert the host immune response is to cause phagosome rupture and escape into the cytosol (termed cytosolic translocation). ESAT-6 (EsxA) has been implicated in Mtb translocation to the cytosol [198,336,337]. EsxA is secreted as a heterodimer with CFP-10 (EsxB) through the ESX-1 Type 7 secretion system [75]. The Mtb genome encodes for 5 unique Type 7 secretion systems (ESX-1 to ESX-5), and among these, ESX-1 is the most studied. Several components and substrates of the ESX-1 secretion system are Mtb virulence factors (Table 2). It should also be noted that other Mtb secreted proteins not directly secreted via the ESX-1 machine can access the cytoplasm via the ruptured phagosome [305] or because the bacteria now reside in the cytoplasm.

As the canonical virulence factor of the ESX-1 secretion system, EsxA has been implicated in a variety of virulence activities [248]. EsxA can bind the cell surface of epithelial cells via laminin [338], M cells via scavenger receptor B1 [43] and macrophages via β2-microglobulin [339] and TLR2 [340] to mediate activities like cell entry [43,338] and immunomodulation [339,340]. Once Mtb is internalized, EsxA facilitates phagosome rupture [341] which results in activation of the cytosolic surveillance pathway and induction of IFN-β [342]. Membrane binding and lysis occur when EsxA dissociates from EsxB under acidic conditions [343] typically found in late phagosomes, in a process requiring EsxA Nα-acetylation [344]. EsxA membranolytic activity is enhanced by PDIM [345] and can be modulated by single amino acid substitutions at glutamine 5 of the EsxA N-terminus [249]. Interestingly, mutants in two conserved residues in the unstructured C-terminal tail of EsxA which are required for phagosome rupture and virulence maintain the ability to lyse acidified liposomes, suggesting that virulence activities and membrane lysis can be decoupled [346].

While the majority of focus has been on the impact of Mtb ESX-1 and EsxA on membrane rupture, another Mtb protein, Rv3091, was recently shown to allow phagosome escape of an avirulent organism, *M. smegmatis* [268]. Rv3091 is homologous to patatin-like phospholipases (PLPs) that are essential virulence factors of *Rickettsia typhi* [347,348] and *Legionella pneumophila* [349,350]. Indeed, Mtb Rv3091 has PLP activity [268], suggesting that it may serve a similar function in Mtb pathogenesis as for *R. typhi* and *L. pneumophila*, though studies in Mtb have yet to be reported.

### Subversion of host autophagy

Autophagy is an evolutionarily conserved process mediating degradation of intracellular organelles and proteins during cell differentiation and stress. Autophagy can be harnessed by eukaryotic cells as a host defence against intracellular pathogens and in this context is termed xenophagy. During xenophagy, intracellular organisms are ensnared within autophagosomes that eventually fuse with lysosomes forming autolysosomes [203,351]. The first report of a role for autophagy in mycobacterial pathogenesis showed that autophagy is engaged in macrophages in the context of infection with BCG [352]. Subsequent results demonstrated that the Mtb ESX-1 machine (which is absent in the BCG strain [353]) is necessary for autophagy activation [124]. Importantly, Mtb xenophagy requires the activity of a variety of host molecules including cGAS/STING [122–125] and the ubiquitin ligases Parkin [354] and Smurf1 [355] that target Mtb for degradation via ubiquitination. Ubiquitination of Mtb or Mtb-containing phagosomes leads to recruitment of autophagy adaptors such as p62 and NBR1 [354,355] which then mediate autophago(lyso)some formation [124,354–357]. In response, Mtb has evolved multiple strategies to counteract autophagic clearance during infection [351], some of which are discussed below.

Because the autophagy process requires the concerted activity of several proteins involved in vesicular trafficking, Mtb virulence factors that target the autophagy machinery overlap with those involved in phagosome maturation arrest. For example, the Mtb proteins SapM and PknG, discussed above, also function to modulate autophagy. SapM interacts with host Rab7 to inhibit autophagosome-lysosome fusion [358]. PknG has the unusual activity of both enhancing autophagy induction but inhibiting autophagosome maturation [258]. It enhances autophagy induction by binding to the pleckstrin-homology domain of AKT, preventing activation of the PI3K-AKT-mTOR pathway and resulting in autophagy induction [258]. Counteracting this activity, PknG also blocks RAB14-GTP hydrolysis preventing Mtb-containing autophagosomes from maturing in autolysosomes, with the net effect of blocking autophagic flux and improving bacterial survival [258]. Highlighting the importance of the PI3K/AKT/mTOR pathway, another Mtb virulence factor known as EIS disrupts autophagy in macrophages [270,271] by indirectly impacting this pathway [271]. EIS increases acetylation of histone H3, which up-regulates IL-10 expression, and IL-10 then activates the PI3K/AKT/mTOR pathway, thus suppressing autophagy. Finally, EspB, an ESX-1–secreted protein, suppresses IFN-γ-induced autophagy in mouse macrophages in part through the downregulation of IFN-γ-receptor [273]. Taken together, the autophagy pathway appears to be a high-value target for Mtb.

### Modulation of cell death pathways

Multicellular organisms are privileged in their ability to sacrifice individual cells for the greater good of the host. There exist several routes to cell death, including apoptosis, pyroptosis, necrosis, and ferroptosis, and Mtb, as an intracellular pathogen, has evolved ways to modulate these pathways. Given their importance to both host and pathogen, these pathways have been extensively studied. Much like the case of cytokine regulation, examples exist of Mtb virulence factors that either inhibit or induce cell death. Here, we will discuss some representative virulence factors that demonstrate the breadth of proteins that target these essential pathways. An exhaustive discussion is, however, beyond the scope of this review.

Apoptosis, or programmed cell death, can be detrimental to Mtb pathogenesis by eliminating its intracellular niche [359] and facilitating antigen presentation by cross-presentation [360]. Various Mtb virulence factors inhibit apoptosis, such as NuoG [279], Ndk [361], PknE [282], Rv3655c [281], Rv3654c [281], and Rv3033 [284]. Macrophages infected with *Mycobacterium kansasii* expressing NuoG had reduced apoptosis whereas infection with Mtb lacking *nuoG* demonstrated increased apoptosis [279]. NuoG was further shown to inhibit the extrinsic apoptosis pathway mediated by TNF-α via neutralization of NOX2-derived ROS [362]. In addition to macrophages, NuoG also inhibits apoptosis in neutrophils and dendritic cells hampering Mtb cell-to-cell spread and delaying the onset of adaptive immunity [280]. Like NuoG, Ndk inhibits apoptosis in macrophages via a mechanism dependent on the neutralization of NOX2 activity [361]. In contrast to the inhibitory roles ascribed to NuoG and Ndk, several virulence factors have been identified that induce apoptosis, including the heparin-binding hemagglutinin antigen (HBHA) [274] and the ESX-1-secreted substrate protein EspC [278]. Whether inhibition or activation of apoptosis by Mtb is more favorable is not yet clear, and may depend on the cell type infected, the growth phase of the bacteria or local environmental conditions.

Another form of cell death that appears to mainly restrict Mtb growth is pyroptosis. Pyroptosis is a programmed necrosis that occurs primarily in myeloid cells such as macrophages [363] that is triggered by the caspase 1/4/5/11-dependent pathway, resulting in inflammasome activation and, consequently, the release of IL-1β and IL-18 [364]. The Mtb protein Rv3364c, a serine protease inhibitor, translocates into the host cytosol where it inhibits cathepsin G activity. This interaction hampers caspase-1 activation and prevents macrophage pyroptosis [293]. More recently, it was found that the Mtb secreted effector protein PknF, a serine/threonine phosphokinase, inhibits the NLRP3 inflammasome dampening pyroptosis activation independently of the ESX-1 secretion system [294]. Another Mtb protein, the zinc metalloprotease Zmp1 may also function to inhibit pyroptosis by inhibiting inflammasome activation [291], though another study did not demonstrate a role for Zmp1 independent of the ESX-1 effector EsxA [365]. More recently, Zmp1 was shown to bind to a mitochondrial respiratory chain complex 1 protein, GRIM-19 [366]. Consistent with the early finding that Zmp1 inhibits pyroptosis [291], loss of GRIM-19 or expression of Zmp1 leads to loss of mitochondrial membrane potential, increased mitochondrial reactive oxygen species and NLRP3 activation [366]. Much like its variable impact on apoptosis, Mtb can also induce pyroptosis in host cells to enhance its survival. For example, Mtb PPE60 induces pyroptosis in human THP-1 cells [225]. In addition, Mtb causes plasma membrane damage in an ESX-1-dependent manner, triggering pyroptosis in THP-1 cells [367]. These results suggest that pyroptosis induction in macrophages by Mtb could be advantageous to the bacteria because it can facilitate Mtb infection of neighboring, uninfected cells. To that end, it is tempting to hypothesize that Mtb temporally regulates host cell pyroptosis (and possibly other cell death pathways) to first establish an intracellular niche for replication by inhibiting pyroptosis (and cell death), and then once that is achieved, to then escape and spread by activating it.

Studies have demonstrated that induction of necrosis, an uncontrolled cell death, favors the survival of Mtb in the host cell [368,369]. CpnT, an outer membrane channel protein in Mtb, also secretes its C-terminal domain, known as tuberculosis necrotizing toxin (TNT) [285,286]. Once it reaches the host cytoplasm through the combined activities of Mtb ESX-1, ESX-2, ESX-4, and ESX-5 Type VII secretion systems [370,371], TNT hydrolyzes NAD+ and induces macrophage necroptosis with associated mitochondrial depolarization [285,372]. Curiously, while CpnT/TNT are sufficient to induce necroptosis in mouse and human macrophages, CpnT is not required for Mtb pathogenesis in C57BL/6 mice [286]. Instead, it has been suggested that CpnT can participate in Mtb dissemination and reactivation *in vivo* [286]. Another possible explanation is that Mtb uses redundant pathways to induce necrosis or necroptosis in the host [75]. To that end, it has been reported that PE11 [288–290] and the protein complex PE25/PPE41 [373] induce necrosis in the host by unknown mechanisms.

It has also been demonstrated that ferroptosis, a type of regulated necrosis dependent on free iron and lipid peroxides accumulation, which is modulated by Mtb showed that ferroptosis induction favors Mtb replication in the host [374]; however, no studies have described Mtb virulence factors responsible for inducing this cell death pathway yet.

## Outlook

In 2015, the WHO launched the “End TB Strategy,” an ambitious plan to eliminate TB incidence by 90% and mortality by 95% by the year 2035 [375]. However, the extended duration required for anti-TB chemotherapy and the emergence of MDR/XDR-TB pose significant challenges toward achieving those goals. Until tests with greater sensitivity and specificity to identify those with ATB or LTBI that will progress to ATB become available, TST and IGRA are the primary immunologic tests for exposure to Mtb. In addition, the 100-year-old BCG vaccine remains the only licensed TB vaccine [376]. One hope is that the study of Mtb virulence mechanisms may provide new avenues for the development of anti-TB therapies and vaccines that will revolutionize the prevention, diagnosis, and treatment of TB to achieve the targets of the WHO End TB strategy.

Although improved diagnostics and chemotherapy are crucial to achieve the milestones proposed by the End TB Strategy, a better vaccine is urgently needed to prevent TB-dependent morbidity and mortality, especially in vulnerable populations at high risk of developing ATB. Currently, about 16 TB vaccine candidates are at various clinical trial phases, including live-attenuated Mtb, killed Mtb, viral vectored and protein/adjuvant-based candidates [377]. A novel vaccine candidate M72/AS01_E_ showed promising results in a Phase 2b trial after 3 years of follow-up in adults from South Africa, Kenya, and Zambia, with a vaccine efficacy in preventing ATB of 50%, the first time in a century that a vaccine against TB has had significant efficacy [378]. Another vaccine candidate, MTBVAC, is based on an attenuated Mtb clinical isolate genetically engineered to stably delete the *phoP* and *fadD26* genes, thus interfering with transcription, synthesis, and/or secretion of multiple virulence factors, including ESAT-6 and PDIM. In contrast to BCG, however, MTBVAC conserves most of the T cell epitopes described for TB including ESAT-6 and CFP-10 [379,380]. Preclinical studies in small animal models and BCG-naive adult rhesus macaques showed that MTBVAC is safe, with high protection against Mtb challenge when compared to BCG [379,381]. MTBVAC has successfully entered a multi-center Phase 3 efficacy trial in newborn babies in South Africa, Madagascar, and Senegal [382]. Finally, after the remarkable success of the mRNA-containing lipid-particle SARS-CoV-2 vaccines, this promising platform has been adapted to TB vaccines with the intent to deliver mRNA molecules encoding Mtb surface molecules into host cells using lipid nanoparticles. Indeed, clinical trials of an mRNA vaccine for TB are set to begin this year, according to a BioNTech Press Release [383]. Leveraging advances in our understanding of Mtb virulence and pathogenesis with new delivery and adjuvant technologies has the potential to dramatically alter the trajectory of TB prevention.

Newer TB antigen-based skin tests (TBSTs) have been developed to measure the immune response to Mtb specific antigens, many of which are also Mtb virulence factors. Emerging evidence suggests that TBSTs tests are cost-effective, accurate, offer similar specificity to IGRA, and may provide more reliable results in immunosuppressive patients, such as people living with HIV [384]. Regarding IGRAs, other mycobacterial virulence factors can also be used to improve their specificity and sensitivity, including EspC (an ESX-1 substrate), EspF (an ESX-1 associated protein), and *Rv2348-B* (unknown function), to identify individuals that do not react to ESAT-6 or CFP-10 [385]. Finally, as discussed above, immunologic-based tests cannot definitively identify individuals infected with living Mtb. Recently, novel glycan-based dyes [386] and positron-emission-based probes [387] that take advantage of the unique components of the mycobacterial cell wall have been developed that can visualize living Mtb *in vitro* or *in vivo*, respectively. These or other compounds targeting either bacterial growth-specific or host inflammation-specific processes [388] hold promise as cutting-edge diagnostics to reduce diagnostic uncertainty and shorten treatment delays associated with our currently available tools.

Despite recent advances in TB drug development, additional antibiotics and shorter treatment regimens are necessary to prevent the emergence of drug-resistant strains. While traditional approaches toward developing directly acting antimicrobials have yielded several new classes of therapeutics [31,389], targeting bacterial virulence pathways and their host responses holds promise as the next frontier of Mtb drug development [390]. Thus, targeting critical virulence lipids [391] or virulence factors [392] could yield unique therapeutics not identified by traditional methods. Alternatively, whether by enhancing autophagy [393], reversing Mtb-induced blockade of phagosome maturation [394] or balancing the host inflammatory response [395], developing approaches that direct the host immune system to overcome its “fundamental immunodeficiency” [396] that licenses Mtb to successfully replicate will help end the world’s longest-lasting endemic pandemic.
